# Semantic relatedness and the efficacy of retrieval practice

**DOI:** 10.1038/s41539-026-00416-8

**Published:** 2026-04-04

**Authors:** Mohan W. Gupta, Steven C. Pan, Timothy C. Rickard

**Affiliations:** 1 Department of Psychology, Princeton University, Princeton, NJ USA; 2 Department of Psychology, National University of Singapore, Singapore, Singapore; 3 Department of Psychology, University of California San Diego, San Diego, CA USA

**Keywords:** Neuroscience, Psychology, Psychology

## Abstract

Retrieval practice (i.e., practice testing) enhances recall relative to other study methods, but it is still unclear when it helps most. Here, we ask if testing is more beneficial for weakly or strongly related word pairs. Participants studied word pairs with either low or high semantic relatedness, then practiced them using either test-with-feedback or restudy. We gave a final cued-recall test 24 hours later. Rather than relying on an ANOVA interaction on proportion correct, which can be hard to interpret when the link between latent memory strength and accuracy is unknown, we evaluated performance using a principled reference model and used cumulative distribution matching. Testing improved recall in both groups, but its benefit was 26% smaller for highly related pairs. This pattern was not explained by ceiling constraints, either across participants or at the item level. The model’s characteristic quadratic relation between test and restudy performance held, but the high-relatedness data showed a systematic reduction in the incremental gain from testing. One interpretation consistent with the model is that study and test memory strengths become positively correlated for highly related materials. These results clarify how semantic structure shapes the testing effect and when practice testing is likely to pay off most.

## Introduction

The testing effect (TE)—a phenomenon wherein retrieval-based learning enhances episodic memory more than restudy—has been extensively studied and is well-documented across multiple experimental designs, including variations in test type, material type, feedback condition, retention interval, training sequence, and levels of prior learning^1–10^. Although the TE is primarily considered an episodic memory phenomenon, there has been substantial interest in the role of semantic memory in the effect^11–20^.

Only a few studies, however, have directly investigated whether the efficacy of testing relative to a restudy control condition (henceforth, the relative efficacy of testing) varies as a function of semantic relatedness. A meta-analysis of the retrieval practice literature by Rowland (2014) found that the magnitude of the cued recall TE did not significantly differ when the stimulus materials were cue-target pairs that are related (Hedges’ *g* = 0.66) versus unrelated (*g* = 0.67). The amount of data available for that analysis, however, was acknowledged to be limited. Further, those effect sizes were estimated in the context of unmatched, between-study differences in relatedness rather than through controlled experiments.

To our knowledge, there are only two contemporary papers (c.f.^21^,) in which cue-target relatedness was manipulated within experiments. Carpenter (2009) conducted two experiments in which study and training phase materials were word pairs with high or low relatedness. During training, pairs were either restudied or tested using cued-recall without feedback, and the final test involved free-recall of the target word from each pair. In neither experiment was there an effect of relatedness on final test performance in the restudy condition. In Experiment 1, the TE on the final test was smaller for high relatedness pairs, suggesting that the relative efficacy of testing may be weaker in that case. In Experiment 2 there was no relatedness effect on the TE. Walsh and Rissman (2023) compared the TE for low and high relatedness word pairs using cued recall without feedback in both the training and final test phases. In the overall data, they observed no statistically significant difference in the TE magnitude across the two relatedness levels. In a secondary analysis, they observed a smaller TE in the high than in the low relatedness condition when only including test items for which a correct response was made during the training phase. However, the interpretation of that analysis is complicated by the fact that final test proportion correct for the full set of restudied items was compared to final test proportion correct for the subset of test items that were answered correctly on the training phase test.

**Fig. 1:**
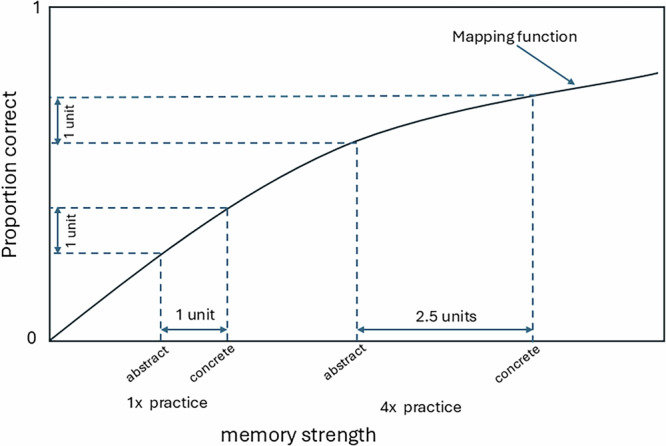
Uninterpretable interactions. Depiction of a non-linear mapping between a psychological construct (memory strength) and a dependent measure (proportion correct).

For current purposes, a limitation of the Walsh and Rissman (2023) and Rowland (2014) studies is that the interactions in proportion correct between training task (restudy vs. testing) and relatedness level are uninterpretable. With the exception of special cases (most notably a crossover interaction), if the quantitative mapping between the underlying psychological processes of interest and the observed dependent variable is not known, then an observed interaction (or lack there-of) in the dependent variable is theoretically uninterpretable. Two prior papers have focused extensively on the issue of uninterpretable interactions^22,23^. As an illustration of this problem in a memory context different from that of the present work, consider an experiment using word pairs with factors of word type (concrete or abstract) and study repetition (1x or 4x). After the study phase, there is a cued recall test for each pair. Figure 1 depicts a plausible non-linear mapping between the psychological construct of memory strength and the observed proportion correct on the final test. In this example, the difference in memory strength for abstract vs. concrete pairs is larger following 4x study (2.5 units of change) than following 1x study (1 unit). Hence, there is an interaction at the psychological level. However, the non-linear quantitative mapping yields no interaction in the observed proportion correct. Generally, if the research goal is to understand psychological processes (rather than to draw purely applied inference), and if the quantitative mapping function from the psychological construct to the dependent variable is unknown and cannot be modeled successfully for at least the two levels of one of the variables (as in the present work), then the observed interaction in the dependent measure is in most cases not interpretable. The same reasoning holds if there is no interaction at the psychological level but there is an interaction at the level of the dependent measure.

Given the substantial interest in the role of semantic memory in the TE, methods for circumventing the problem of uninterpretable interactions are needed. In the current paper we use two approaches^22^: (1) application of an empirically supported model for low relatedness materials as a yardstick against which to evaluate the relative efficacy of testing for high relatedness materials, and (2) application of a variant of the matching strategy – in the present case, matching groups on restudy performance and determining whether there are testing effect differences—in the context of cumulative proportion correct distribution analysis. In the next two sections we summarize such a model, the dual-memory model of test-enhanced learning, and the empirical work supporting it. We then describe three hypotheses about the effect of semantic relatedness in the context of that model.

**Fig. 2:**
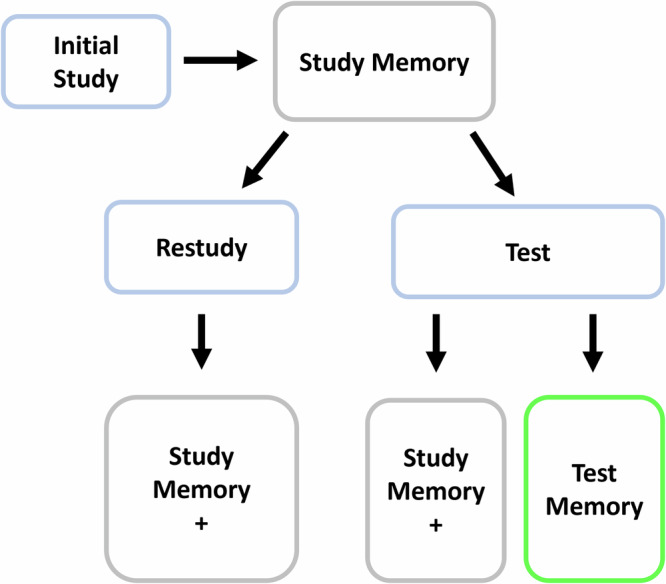
Conceptual explanation of the dual memory model. A simplified depiction of the dual memory model that highlights the core claim. On initial study, a study memory is created. During restudy, that memory strengthened (+). Testing is a sufficient event to create a second memory, test memory, as well as strengthen the initial study memory. These two routes of retrieval compose the benefit of testing relative to the single route of retrieval of restudy.

Here we summarize aspects of that model that are central to the current work, drawing on previous work^4,9,24^. The core claim of the dual-memory framework (Fig. 2) is that, whereas restudy strengthens the memory retrieval route that is formed during initial study (*study memory*), a test with feedback trial both strengthens study memory and forms a separate memory of the test event (*test memory*), yielding two independent retrieval routes for the tested items on the final test (through study memory and through test memory). Here and elsewhere, the strength of a memory is positively related to probability of recall of the target (given the cue) through a specified route at some later time.

More specifically, on the first test trial following an initial study trial, the presentation of the cue creates a new episodic memory of that cue in the context of the goal to retrieve the target–what we refer to as *cue memory*. The target can then be retrieved in one of two ways: from the initially encoded *study memory* from study phase or from the *test memory*, which is created through retrieval in the training phase, regardless of retrieval accuracy, provided that correct answer feedback is provided (henceforth, feedback). During test trials with feedback, study memory is also strengthened—either because successful retrieval reinforces the association acquired during the study phase or because feedback supplies the correct target on an initially incorrect trial. The strength of a test memory does not depend on whether the target was correctly retrieved on the test trial, an assumption that is supported by prior work^9,25^. It is further assumed that incorrect target retrieval in the context of feedback does not affect learning or retention^26^.

To facilitate development of a quantitative model that is consistent with the framework outlined above and that makes precise and testable predictions, Rickard and Pan (2018) made three simplifying assumptions regarding training phase learning. Assumption 1 is that study memory strengths after training (and on the final test) are identically distributed for restudied and tested-with-feedback items. This assumption is plausible if we stipulate that (1) study memory strengthening occurs whenever the cue and target are simultaneously active in working memory (as during restudy and feedback processing) and (2) the duration of that joint activation is not a critical factor in learning (at least beyond some minimal duration). The assumption is consistent with our findings in prior work of equivalently good dual memory model fits using 1 s and 2 s of feedback. Assumption 2 is that study and test memory strengths are identically distributed. Assumption 3 is that study and test memory strengths for tested items are stochastically independent. These assumptions are made on the basis of simplicity and we do not necessarily expect them to hold in the general case.

Given a few more secondary assumptions, Rickard and Pan (2018) showed that, for a given participant and item, probability correct in the test condition of the final test (P­_T_) is given by the probabilistic inclusive-or equation for the case of stochastic independence:

1 $${{\rm{P}}}_{{\rm{T}}}={{\rm{P}}}_{{\rm{T}}-{\rm{s}}}+{{\rm{P}}}_{{\rm{T}}-{\rm{t}}}-{{\rm{P}}}_{{\rm{T}}-{\rm{s}}\,}{{\rm{P}}}_{{\rm{T}}-{\rm{t}}}$$

where P_T-s_ is the probability of correct retrieval through study memory for a randomly selected item in the test condition, and P_T-t_ is the probability of correct retrieval through the test memory for a randomly selected item in the test condition.

As indicated by the foregoing discussion, P_T-s_ is assumed to equal P_T-t_ in that equation. It also follows from the model description above that probability of correct retrieval through study memory in the restudy condition (P_R_) is the same as the probability of correct retrieval through study memory in the test condition (P_T-t_). Hence, in this simplest case quantitative instantiation of the theoretical framework, P_T-s_ = P_T-t_ = P_R_. Equation(1) can thus be reduced to,

2 $${{\rm{P}}}_{{\rm{T}}}={{\rm{P}}}_{{\rm{R}}}+{{\rm{P}}}_{{\rm{R}}}-{{\rm{P}}}_{{\rm{R}}}* {{\rm{P}}}_{{\rm{R}}}=2{{\rm{P}}}_{{\rm{R}}}-{{{\rm{P}}}_{{\rm{R}}}}^{2}$$

and the equation for the TE is,

3 $$\mathrm{TE}=({2{\rm{P}}}_{{\rm{R}}}{\rm{\mbox{--}}}{{{\rm{P}}}_{{\rm{R}}}}^{2})-{{\rm{P}}}_{{\rm{R}}}={{\rm{P}}}_{{\rm{R}}}-{{{\rm{P}}}_{{\rm{R}}}}^{2}$$

The model predicts that the probability correct in the test condition (P_T_) and the TE magnitude are both a function solely of probability correct in the restudy condition, P_R_. To a close approximation, the predictions of Eqs.(2) and (3) generalize to subject-level proportion correct without modification:

4 $${{PC}}_{T-{predicted}}={2\,* \,\mathrm{PC}}_{{\rm{R}}}-{{\mathrm{PC}}_{{\rm{R}}}}^{2}$$

Thus, the predicted TE according to the model the value of PC_T-predicted_ minus PC_R_:

5 $${{TE}}_{{predicted}}={\mathrm{PC}}_{{\rm{R}}}-{{\mathrm{PC}}_{{\rm{R}}}}^{2}$$

The model makes PC_T_ and TE predictions independently for each participant and with no free parameters. Although a model with no free parameters surely has boundaries in applicability, it has proven useful as an initial quantitative implementation of the dual-memory framework when applied to the low relatedness materials and the cued-recall TE design that is used in the current paper.

Rickard and Pan (2018) showed that the quantitative model closely predicts proportion correct in the test condition, and hence the TE, across 512 participants from multiple cued recall experiments involving different material types (word pairs, triplets, and history and biology facts) and retention intervals (1, 2, and 7 days). They showed that the model provides a viable account of the TE for the cases of both feedback and no feedback during training.

**Fig. 3:**
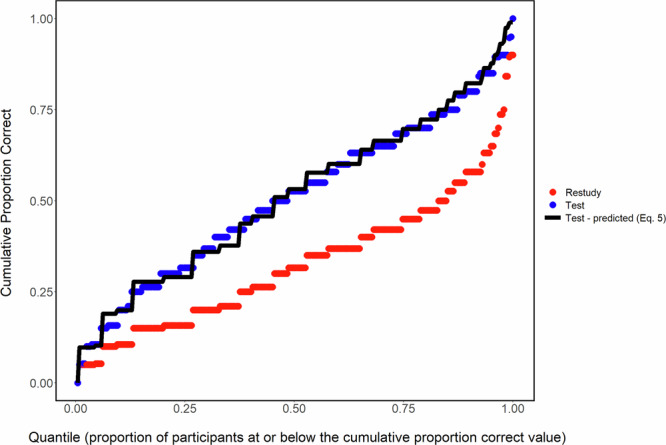
Dual memory model fits to data. Cumulative distribution graph of the combined data from Experiments 1 and 2 of Pan et al. (2015). The Test-predicted (black) line is the dual memory model prediction Eq.(5). The test-predicted proportions closely match the observed proportion across nearly the entire distribution.

Cumulative distribution plots show that the model’s predictions hold not only for mean proportion correct but also for the proportion correct distribution (Rickard, 2020). An example cumulative plot and model fit to word-pair data from Pan et al. (2015; Experiments 1 and 2 combined) is shown in Fig. 3. In that plot, PC_R_ is sorted from smallest to largest across all participants. The PC_R_ and PC_T_ values are also independently sorted and plotted from smallest to largest. There are 242 equally spaced quantile values on the x-axis, scaled from zero to one, representing each of the 242 participants. Hence, in this type of plot, a given participant’s PC_R_ and PC_T_ values would occur on the same quantile only by chance. Finally, the PC_T-predicted_ value at each quantile is the Eq. (1) model prediction, based on the PC_R_ value at the same quantile (for further discussion, see Rickard, 2020).

We considered three plausible scenarios for the effect of relatedness that can be framed within the dual-memory model. In scenario 1, the relative efficacy of testing is the same across relatedness levels (low and high). In the dual-memory model, this scenario would correspond to equivalent expected study and test memory strengths over items in both the low and high relatedness group. Overall memory strengths and associated final test proportions correct are expected to be higher in the high relatedness group, but study and test memory strengths would remain equivalent within each group. In this case, the model predictions Eqs.(1) and (2) will hold for both groups. This scenario defines equivalent testing efficacy for low and high relatedness groups in the context of the model.

Because of the theoretically predicted (and empirically established) quadratic relationship between restudy and test proportion correct, Scenario 1 does not imply that there must be equivalent *TE magnitudes* in the two groups (for this and related reasons, we differentiate in the current work between the TE magnitude as a proportion correct difference score and the relative efficacy of testing). Rather, TE magnitudes in this scenario may be the same in the two groups or may be different, depending solely on PC_R_ values. If, for example, a typical participant in the low relatedness group has PC_R_ = 0.3, then the predicted TE for that participant is 0.21 Eq.(1), and if a typical participant in the high relatedness group has PC_R_ = 0.7, then the predicted TE is also 0.21. In this case, there would be no interaction in an ANOVA on observed proportion correct with factors of relatedness level and training task. Conversely, if a typical participant in the low relatedness group has PC_R_ = 0.5 (yielding a predicted TE of 0.25), and a typical participant in the high relatedness group has PC_R_ = 0.8, (yielding a predicted TE is 0.16), then there would be an ANOVA interaction, even though in the model study and test memory strengths remain equivalent within each group and hence the relative efficacy of testing remains the same. For this reason, an ANOVA interaction test can be misleading and is not a reliable method for assessing whether relatedness affects testing efficacy.

This discussion illustrates the general problem of theoretically uninterpretable interactions^22,23^, wherein an interaction in the observed dependent measure does not necessarily indicate an interaction in the underlying psychological processes of interest, and conversely, when the absence of an interaction in the dependent measure does not necessarily indicate an absence of interaction at the process level. One preferred approach in this case is to not rely on the ANOVA interaction test, but rather to evaluate fits of an empirically supported theoretical model, such as the dual-memory model as applied here. A good fit of that model to the means of both groups would support Scenario 1, regardless of whether the TE magnitudes for the two groups are the same or different. We also applied a second, complementary strategy for addressing the interaction problem (a variant of the matching strategy^22^) in which the cumulative proportion correct distributions were used to determine whether the TE magnitudes of each group match the model prediction across all levels of restudy proportion correct.

Scenario 2 is that high semantic relatedness reduces the efficacy of testing. In this case, the model prediction should overestimate the mean TE magnitude in the high relatedness group, and the TEs at all matched levels of PC_R_ in the cumulative distributions should be smaller than predicted. The third scenario is that high semantic relatedness increases the efficacy of testing relative to restudy. In this case, the model prediction should underestimate the mean TE magnitude for the high relatedness group, and the TEs at all matched levels of PC_R_ in the cumulative distributions should be larger than predicted.

## Results

### Mean final test performance and model fits

**Fig. 4:**
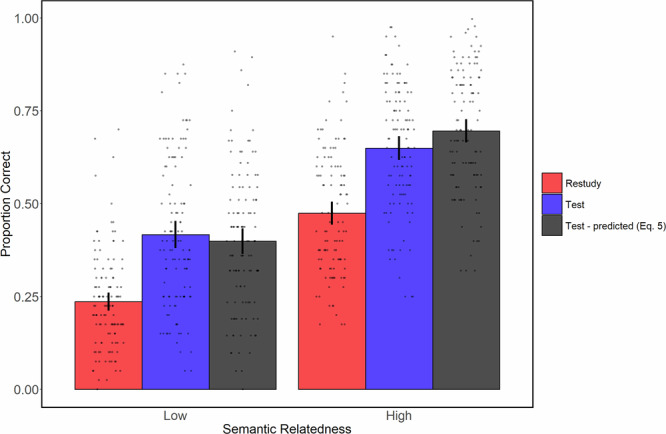
Dual-memory model does not fit for highly related items. Final test means along with independent strengths dual-memory model predictions for the test condition for each relatedness group. Error bars are 95% CIs, calculated independently for each condition.

Final test results for proportion correct means, as well as the model predictions for the test conditions means, are shown in Fig. 4. A mixed factors ANOVA on the observed proportion correct (setting aside the predicted PC_T_), with factors of Training Task (restudy vs. test; within participants) and Relatedness (low vs. high relatedness; between participants), produced a significant (at α = 0.05) main effect of Training Task, *F*(1, 229) = 514.1, *p* < 0.0001, *η*_*p*_^2^ = 0.69, BF_10_ = 5.91e^56^, a significant main effect of Relatedness, *F*(1, 229) = 126.6, *p* < 0.0001, *η*_*p*_^2^ = 0.36, BF_10_ = 3.01e^20^, and a non-significant interaction, *F*(1, 229) = 0.108, *p* = 0.74, *η*_*p*_^2^ = 0.0005, BF_01_ = 6.86.

The absence of a significant interaction between Relatedness and Training Task replicates the full dataset results of Walsh and Rissman (2023) and Experiment 1 of Carpenter (2009), as well as the meta-analytical results reported in Rowland (2014). However, a conclusion of equivalent relative testing efficacy is not warranted, because the absence of an ANOVA interaction is in this case uninterpretable. It does not necessarily indicate the absence of interaction at the psychological process level; that is, it does not demonstrate that the relative efficacy of testing is the same at low and high relatedness levels.

A preferred, model-based approach in this scenario is to test for an ANOVA interaction in a comparison of observed vs predicted test performance, with factors of Data Type (observed PC_T_ vs. PC_T-predicted_; restudy data were excluded from this analysis) and Relatedness. The ANOVA yielded a non-significant main effect of Data Type, *F*(1, 229) = 2.89, *p* = 0.091, *η*_*p*_^2^ = 0.01, BF_01_ = 2.67, a significant main effect of Relatedness, F(1,229) = 135.7, *p* < 0.0001, *η*_*p*_^2^ = 0.37, BF_10_ = 3.47e^21^, and most critically, a significant interaction between Data Type and Relatedness, *F*(1, 229) = 16.2, *p* < 0.0001, *η*_*p*_^2^ = 0.07, BF_10_ = 204.9. Pairwise t-tests were performed on the simple effects of Data Type. There was a non-significant difference in the low relatedness group, *t*(117) = 1.42, *p* = 0.16, BF_01_ = 3.65, but a robust difference in the high relatedness group, *t*(112) = –4.74, *p* < 0.0001, BF_10_ = 2411. In summary, there was evidence from the model fits that the efficacy of testing is reduced in the high relatedness group compared to the low relatedness group.

**Fig. 5:**
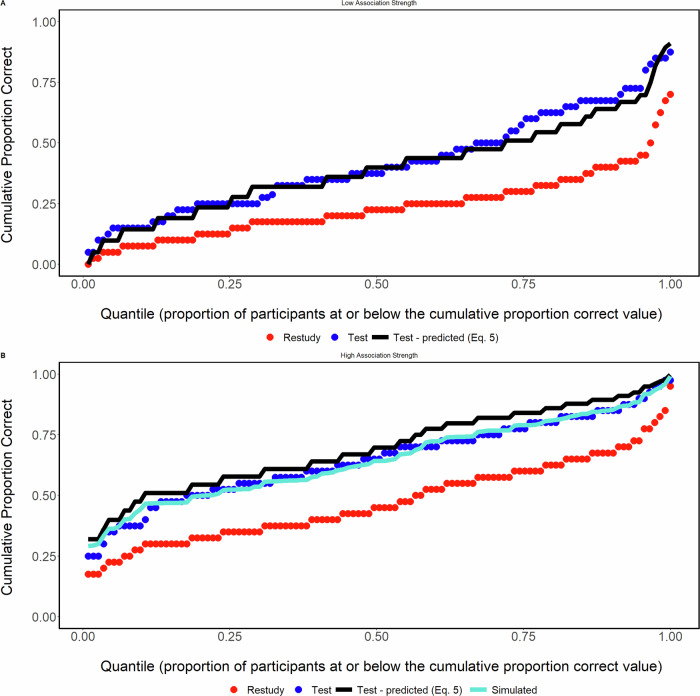
A modified dual memory model fits highly related items. **A** Cumulative distribution plot for the low semantic relatedness group. **B** Cumulative distribution plot high semantic relatedness group, including the fit of the simulated correlated strengths.

Mean final test PC_R_ in the high relatedness group (0.47) is high relative to most of the literature, including the majority of our papers in which the dual memory model predictions were fitted to data. This fact raises the possibility that the failure of the model to fit well for the high relatedness group reflects a fundamental limitation of the model, even in the low relatedness case, when restudy proportion correct is in the range of 0.47. If so, then our results do not necessarily imply lower efficacy of testing in the high relatedness case. However, two sources of evidence argue against that possibility. First, in multiple cumulative distribution analyses of low relatedness data^27^, the model predictions have held across the entire range of restudy proportion correct. An example from prior work is the cumulative distribution shown in Fig. 3. See also the cumulative fit to the current low relatedness group in Fig. 5. Second, the model predictions for low relatedness materials have held in our prior work even when mean restudy correct exceeds 0.47. For example, in Gupta, Pan, and Rickard (2022), increasing initial study phase repetitions to eight produced final test mean restudy accuracy of 0.53, yet the model fit was almost exact. These findings support the validity of our approach of using the dual-memory model as a yardstick for estimating the relative efficacy of testing in the high vs. low relatedness groups.

### Cumulative distributions

The cumulative distribution results are shown in Fig. 5 for both groups, along with the PC_T_ predictions of the dual-memory model for both groups and the prediction of a modified version of that model for the high relatedness group (the correlated strengths version) that will be introduced in the General Discussion as a candidate account of our results. As expected based on prior work for low relatedness materials, the model fitted well across most of the distribution for the low-relatedness group. The predictions for that group deviated from the data primarily in the upper-middle range of distribution, where the model underpredicted the observed PC_T_ magnitude. That form of fit deviation was not observed in prior distribution fits to low relatedness data^4,24,27^. See also Fig. 3 of the present paper. We conclude that the deviations likely reflect sampling variability.

On the other hand, the model systematically overpredicted the PC_T_ values across all levels of PC_R_ for the high relatedness group. Because the PC distributions reflect both proportion correct sampling variability and individual differences in task ability^24,27^, these results indicate that high relatedness materials reduce the efficacy of testing across a broad spectrum of individual differences in task ability. This result rules out the possibility that the mean TE was smaller in the high relatedness group merely because of a proportion correct ceiling effect limited to high performing participants.

The pivotal contribution of the distribution analysis in confirming the lower relative efficacy of testing in the high relatedness group can be appreciated by noting that (1) the full range of possible PC_R_ values was exhibited across participants in both the low and high relatedness groups, and (2) at the great majority of those PC_R_ values the TE magnitude was smaller in the high relatedness group, a result that is reflected in the consistently smaller PC_T_ values in Fig. 5b than were predicted by the model. That strong distribution evidence of lower relative testing efficacy in the high relatedness group may appear incompatible with the small and non-significant difference between the group mean TEs (Fig. 4). However, that result is fully explained by the higher restudy proportion correct in the high relatedness group in the context of the quadratic relation between PC_R_ and PC_T_. In the quadratic relation, the largest TEs occur when PC_R_ is between about 0.4 and 0.6. In the high relatedness group, about 29.2% of participants had PC_R_ values within that range, whereas in the low relatedness group only 10.1% of PC_R_ values were within that range. Hence, the similar mean TE magnitudes in the two groups is the net effect of two opposing factors: (1) the greater efficacy of testing (at matched PC_R_ levels) in the low relatedness group, countered by (2) attenuation of TEs in the low relatedness group due to the smaller percentage of participants with PC_R_ values between about 0.4 and 0.6. Those two independent factors offset one another, masking the interaction between task and relatedness that is present at the psychological process level and yielding roughly equivalent group mean TEs. In summary, the cumulative distribution analysis can be understood as controlling for the quadratic effect and thus revealing the lower intrinsic efficacy of testing in the higher relatedness case.

### Item-level analysis

Although the cumulative distribution analyses indicate no proportion correct ceiling effect across participants in the high relatedness group, it remains possible that there is an item-level ceiling effect that is widely distributed over participants, such that for a subset of items, proportion correct in both the restudy and test condition approached 1. If so, then the smaller TE magnitude in the high relatedness group may not reflect a fundamental effect of relatedness on the efficacy of testing, but rather a methodological limitation. However, two results appear to rule out that possibility. First, the largest item-level proportion correct in the restudy condition of the high relatedness group was 0.79 (for the item “mask – face”) and the largest proportion correct in the test condition was 0.93 (for “report – card”). The great majority of item proportions correct were well below those values, suggesting that there is no pervasive ceiling effect.

In a complementary approach involving all items in the high relatedness group, we performed a generalized linear model analysis using the Proc GLIMMIX procedure in the SAS 9.4 software. The model specified a binary response distribution (“1” for correct or “0” for incorrect) and random intercepts for subjects and items, with training condition (restudy vs test; categorical), item semantic relatedness (continuous), and their interaction, as within-subject predictors. The relatedness measure is based on associative norms for each item as listed in the Supplement. There were significant effects of both semantic relatedness, *F*(1, 8733) = 24.54, *p* < .0001, confirming the expected increase in overall accuracy with increasing item relatedness, and training condition, *F*(1, 8733) = 82.77, *p* < .0001, confirming the testing effect. The interaction, however, was not statistically significant, *F*(1, 8733) = 0.05, *p* = 0.82. Hence, there is no evidence for compression of the TE for higher relatedness items.

## Discussion

The TE proved robust for word pairs with both low and high levels of semantic relatedness, and as expected based on prior work, the dual-memory model fitted well for the case of low relatedness. Nevertheless, the efficacy of testing was reduced by about 26% in the high relatedness group (based on the difference between mean TE in that group and the model’s predicted mean), a result that is most compellingly demonstrated by the smaller than predicted TE magnitude at each level of PC_R_ in the cumulative distribution plots. Accordingly, the two alternative scenarios hypothesized at the outset of this study, in which semantic relatedness has either no effect on relative testing efficacy or amplifies it, appear to be ruled out.

Our null proportion correct ANOVA interaction between training task and semantic relatedness mirrors three prior null interaction results on this topic noted in the Introduction. We have demonstrated in the current work, however, that all of those null interactions are theoretically uninterpretable. Further, our modeling and distribution analyses clarify how a null ANOVA interaction can mask a real interaction between relatedness and testing efficacy. Specifically, even when testing is less effective for high relatedness materials, the quadratic relationship between PC_R_ and TE magnitude can obscure this difference in a traditional ANOVA interaction. More generally, the presence or absence of an ANOVA interaction in any TE experiment is likely to be theoretically uninterpretable whenever both the restudy and test condition mean proportions correct change in the same direction across levels of other experimental factors (e.g., semantic relatedness). In that case, alternative bases for theoretical inference are required, such as the application of a process model and the matching strategy in the current work. More generally, the risk of an uninterpretable interaction is not limited to testing effect or psychology experiments. It may be present in the context of any experiment in which an interaction could occur^22,23^.

Rickard and Pan (2018) suggested that for highly related materials the pre-existing connections in the semantic network may lead to study and test memories that incorporate overlapping information. A plausible consequence of that overlap is positively correlated study and test memory strengths across items for each participant, rather than independent strengths as assumed in the dual-memory model for low relatedness materials. To investigate this possibility, we extended the model through simulation by incorporating a single free parameter, *ρ*, representing the correlation between study and test memory strengths. The simulation details and predictions of that extended model are described in the Supplementary and a visualization of the correlated memory strengths can be found in Supplementary Fig. 1. It is guaranteed that use of *ρ* as a free parameter in that model will provide exact fits to the mean TE for the high relatedness group because it acts as a scaling parameter for the mean PC_T-predicted_. However, *ρ* does not guarantee good distribution fits. The best fit to the mean data was obtained for *ρ* = 0.334. For fits of other *p* values, see Supplementary Fig. 2. And as shown in Fig. 5b, that value of *ρ* yielded a good fit across the bulk of the distribution for high relatedness items.

The correlated strengths model is a straightforward and plausible extension of the dual-memory model to the high relatedness case, requiring only a single free parameter. One plausible psychological basis of this account is that for highly related words, study and test memories are formed by partially overlapping features drawn from pre-existing semantic associations between the cue and target. This shared representational substrate yields shared feature activations across memories, so items that strongly recruit the cue–target semantic scaffold during encoding and practice will have correlated memory strengths. This view predicts that neural pattern similarity between study and test (e.g., representational similarity analyses) should covary with the model-estimated *ρ*, and that item-level semantic similarity (e.g., cosine similarity in word embeddings^28^) should likewise track *ρ*.

The correlated strengths account raises the possibility that the original dual-memory model, which assumes independence of the two retrieval routes, may be incorrect or incomplete for low relatedness materials. After all, low relatedness does not imply zero relatedness, so some degree of dependence between the two retrieval routes may exist. Thus the question arises: why does the independent routes model fit so well in our current and past work to low relatedness materials? The correlated-strengths account makes clear that when the correlation parameter ρ is small, predicted TE magnitudes are nearly indistinguishable from the independence case (Supplementary Fig. 2). In particular, the difference in TE magnitude across the distribution when *ρ* = 0 vs when *ρ* = 0.1 is negligible. It is plausible that for low relatedness pairs *ρ* is <= 0.1, given for the high relatedness group the estimated value *ρ* was 0.334 and that the mean semantic relatedness was more than eight times higher in the high than in the low relatedness group. As such, the good fits of the original independent routes model to low relatedness materials is not necessarily problematic for the dual route framework.

Carpenter (2009) hypothesized that elaborative retrieval—by way of mediating concepts—is more likely to occur on test trials than on restudy trials, accounting for the TE. Carpenter also speculated that mediator links are more likely to form for weakly related cue-target pairs than for strongly related pairs. When cues and targets share a strong preexisting association, retrieval can proceed through direct association, leaving little need for elaboration. In contrast, weakly related pairs require the learner to generate additional, non-target associations that can later serve as mediators to the target during final recall. Our results are consistent with that possibility. There are, however, several challenges to the elaborative retrieval model in the recent literature^14,16,19,29^. Nevertheless, the current findings are at least compatible with a core prediction of that model that the benefits of testing and the formation of mediating links should be strongest when cue–target relatedness is low.

The episodic context model^30^ proposes that test trials are more likely to evoke recall of episodic context that is active during prior study trials, and that the incorporation of that episodic context into test trial learning provides the basis for the TE. Karpicke et al. also suggested that encoding of episodic context on test trials might occur less frequently for highly related materials, because the answer is more likely to be retrievable without having to search for and activate that context. Hence, the episodic context account is potentially consistent with current results. However, by analogy to the elaborative retrieval account, it is unclear in that model what level of relatedness would be needed for that effect to occur.

For the episodic context model more generally, there is mixed evidence. Two types of evidence can support that model: (1) evidence that a test enhances context memory more than does restudy, and (2) evidence that test-enhanced context memory is the basis of the testing effect^31^. Evidence of the first type is mixed. A test appears to facilitate both temporal^32,33^ and spatial^34^ context memory, but there is no evidence that retrieval strengthens voice (male vs. female) context memory^32^. Also, Hong et al. (2019) found negative evidence that retrieval enhances color context memory. There appears to be no work in the literature that speaks directly to the second type of evidence. Further, Gupta et al. (2022) showed that repeated study for each item during the initial study phase did not moderate the relative efficacy of cued recall testing, despite the expectation that repeated study would yield stronger or more varied episodic context.

Our finding that the efficacy of testing is reduced for high relatedness materials raises the issue of its efficacy on the other extreme, for very low or zero relatedness materials. For non-linguistic materials with apparent zero relatedness^35–37^, a complete or near absence of the TE relative to the restudy control has been observed in three studies. The dual-memory model does not anticipate those results, though it could accommodate them if it is assumed that test memory cannot form in the complete absence of cue-response relatedness, in which case the model would predict zero TE. However, that account does not provide insight into why the formation of test memory would require at least some minimal degree cue-target semantic relatedness.

For linguistic materials, in contrast, the cued recall TE appears to hold even for the case of very low relatedness^38^. Most pertinent is recent work on the TE for language translation pairs. de Lima and Buratto conducted 5 experiments using Swahili – Brazilian Portuguese words pairs and found robust TEs. They also reported good fits of the dual-memory model. Abel and Roediger (2017) observed similar effects for Swahili-English translation pairs, and unpublished results from our laboratory also demonstrated good fits of the model to the data from their Experiment 1 and to the no retroactive interference group of their Experiment 2. Those results suggest that the original dual-memory model may hold without modification for linguistic materials across a range from very low relatedness to low and perhaps moderate relatedness, with the correlated strengths version of that model accommodating highly related materials. As a caveat to that conclusion, the experiments of both de Lima and Buratto and Abel and Roediger involved multiple training phase item repetitions. It is not known whether the same results and model fits will hold for translation pairs involving one training phase exposure to each item, as in the current experiment and much of the cued-recall TE literature.

Our manipulation operationalized semantic relatedness via normative cue-target forward associative strength^39^. Although we designed the low and high related word pairs to be broadly comparable on basic lexical properties, the present experiment cannot definitively establish that associative strength is the causal factor behind the reduced efficacy of retrieval practice in the high-relatedness condition. It remains possible that forward associative strength covaries with another item property that is the true driver of the effect. However, we are not aware of an accepted alternative candidate in prior work. Future experiments that orthogonalize forward associative strength from other lexical properties would provide a stronger causal test.

The current work constitutes the first strong experimental demonstration that the degree of semantic relatedness for linguistic materials such as word pairs moderates the learning efficacy of retrieval practice. Specifically, the relative efficacy of testing was found to be lower for high relatedness than for low relatedness materials. A modified version of the dual-memory model provided a good fit to the data distribution for the high relatedness group. Our findings may lead to refined recommendations for educational practice by clarifying when retrieval practice is most beneficial, depending on material relatedness.

## Methods

### Participants

We recruited 242 students from the subject pool at the University of California, San Diego to participate in a laboratory experiment in exchange for partial course credit. All but 11 participants completed both sessions and data from the remaining 231 participants were analyzed. In the final sample there were 118 participants in the low relatedness group and 113 participants in the high relatedness group. An a priori power analysis that was conducted for a previous study in our laboratory (see Rickard & Pan, 2020; Experiment 1) indicated that at least 32 participants per group are needed to have a power of 0.8 to detect a difference in proportion correct between PC_T_ and PC_T-predicted_ on final test of at least 0.05. The current sample size far exceeded that value in an effort to minimize sampling variability in the cumulative proportion correct distributions. Participants provided informed consent via in-person signature. All procedures were approved by the institutional review board of the University of California, San Diego, IRB Protocol #141851 in compliance with the Declaration of Helsinki.

### Materials and design

A cued-recall testing paradigm was used. The materials were 80 English word-pairs in each of two master lists (low and high semantic relatedness, respectively; see Supplementary Table 1). Here, “semantic relatedness” is operationalized as normative cue→target forward associative strength from the Nelson et al. norms. Forty of the low semantic relatedness word pairs were identical to those used by Rickard and Pan (2020). Forty additional low relatedness pairs were selected from the same normative data^39^ and using the same criteria as described in Rickard & Pan: nouns that were 3–7 letters, 1–3 syllables, high in concreteness (400–700), had frequency of at least 30 per million, and were weakly associated. The mean forward associative strength for these pairs was 0.028. The cue words for high relatedness pairs remained the same as those used for the low relatedness pairs. The 80 new target words were selected with the same criteria as for low relatedness pairs, with the exception that the target word selected for each cue was that with the highest associative strength in the database (mean = 0.238). For counterbalancing purposes, two versions of each master list were created. In version 1, half of the word pairs were randomly assigned to the test condition and the other half to the restudy condition, and in version 2 those assignments were reversed.

### Procedure

The experiment was conducted in-person using laboratory desktop computers and the E-Prime 2.0 software program. Participants were randomly assigned to either the low or the high relatedness group and the training phase task (restudy vs. testing with feedback) was manipulated within-participants. The two versions of the master list for each group were counterbalanced using odd versus even participant numbers. Word pairs were presented horizontally at the center of the computer monitor, with left- or right-side word placement held constant across all experiment phases. On all test trials, the left-side word was the cue. Word pair presentation order was randomized in each phase, independently for each participant. In the training phase, restudy and test trials were randomly intermixed, a design that has been demonstrated to eliminate the forward testing effect confound during training^24^.

In the study phase of session one, participants in each group passively studied all 80 word-pairs one at a time for six seconds per trial, for a total of 80 trials. Each trial in the subsequent training phase lasted six seconds. In the restudy condition, each word-pair was restudied for six seconds. In the test condition, the cue-word was presented for five seconds, within which time the participants were asked to type the corresponding answer. Both the cue-word and the correct target were then presented for one second, constituting feedback. There was one trial for each word pair, yielding 80 total trials. The final test was administered in session two after a 24-h. delay. It was self-paced and involved one cued-recall test trial per item, with no feedback. If a participant was unable to recall the target-word, they were allowed to advance to the next cue-word without making a response.

### Scoring and statistical analyses

We applied a scoring procedure wherein a response was counted as correct only if the target word spelling was entirely correct. Formal analyses involved *t* tests and ANOVAs, along with Bayes factors. We used the suggested default Cauchy prior value^40^, with a uniform r (prior) value of 0.707 applied to all Bayes factors tests. To evaluate Bayes factor values, we used Raftery’s criteria^41^, whereby Bayes factors falling within the range of 1–3 were considered weak evidence, 3–20 indicated positive evidence, 20–150 was strong evidence, and Bayes factors exceeding 150 were considered very strong evidence. For ease of interpretation, BF_10_ indicates in evidence favor of the alternative hypothesis and BF_01_ indicates evidence in favor of the null hypothesis.

### Transparency and openness

We report how we determined our sample size, all data exclusions, all manipulations, and all measures in the study, and the study follows JARS (Appelbaum et al., 2018). All data, analysis and simulation code are available at the Open Science Framework (https://osf.io/dyv3h/). This study’s design and its analysis were not pre-registered.

## Supplementary information

Supplementary Information

## Data Availability

All data are available at https://osf.io/t5r2h/. Further information and requests for resources should be directed to and will be fulfilled by the corresponding author, TCR (trickard@ucsd.edu).

## Code availability

Analyses are available at https://osf.io/t5r2h/. Further information and requests for resources should be directed to and will be fulfilled by the corresponding author, TCR (trickard@ucsd.edu).
