# Supplementary material for: Semantic relatedness and the efficacy of retrieval practice
Source: NPJ Sci Learn. 2026 Apr 4;11:33. doi: 10.1038/s41539-026-00416-8 (PMC13230953; doi:10.1038/s41539-026-00416-8)
Supplement: Supplementary file 1 — Supplementary Information [file 41539_2026_416_MOESM1_ESM.pdf]

Supplementary

Cue	Low Target	High Target	Low Forward Strength	High Forward Strength
algae	mold	green	0.014	0.306
angel	wings	heaven	0.041	0.327
ape	mammal	gorilla	0.021	0.185
author	poet	writer	0.028	0.324
barn	stable	hay	0.016	0.189
block	street	building	0.04	0.253
bone	skin	dog	0.02	0.255
cement	brick	hard	0.032	0.213
child	mother	adult	0.03	0.101
coffee	morning	cream	0.025	0.081
curve	shape	road	0.018	0.115
elf	fairy	santa	0.013	0.217
factory	product	worker	0.02	0.152
frame	window	picture	0.014	0.811
game	board	play	0.034	0.25
gate	latch	fence	0.013	0.353
guard	prison	watch	0.024	0.115
hole	circle	ground	0.016	0.173
juice	fruit	orange	0.035	0.655
knife	spoon	fork	0.051	0.327
leak	spill	drip	0.034	0.247
mask	crook	face	0.012	0.194

milk	cookie	cow	0.036	0.388
native	foreign	indian	0.056	0.25
neck	collar	tie	0.036	0.079
object	symbol	thing	0.014	0.412
oil	motor	spill	0.014	0.101
paint	picture	brush	0.036	0.327
pencil	point	lead	0.021	0.14
quarter	dollar	dime	0.061	0.252
report	weather	card	0.015	0.152
-	-	-	0.069	0.338
sound	speaker	music	0.024	0.205
stick	branch	glue	0.067	0.117
suit	armor	man	0.014	0.074
tack	staple	nail	0.024	0.181
taste	touch	food	0.016	0.157
throat	voice	sore	0.039	0.164
vehicle	truck	car	0.013	0.74
yard	field	grass	0.016	0.236
alley	lane	dark	0.021	0.186
angle	corner	geometry	0.02	0.179
attic	cellar	house	0.04	0.132
bar	grill	drink	0.034	0.393
beach	blanket	sand	0.012	0.394
boat	ski	sail	0.016	0.134
cabin	camp	log	0.02	0.415

chain	fence	saw	0.022	0.218
cloth	table	fabric	0.012	0.115
college	student	school	0.035	0.238
dew	damp	morning	0.014	0.155
engine	machine	motor	0.033	0.087
fire	hose	water	0.036	0.115
gallon	half	jug	0.014	0.13
garden	weed	flower	0.026	0.391
group	meeting	friends	0.027	0.061
head	face	hair	0.062	0.186
jelly	grape	toast	0.032	0.128
jury	panel	court	0.021	0.25
lab	science	work	0.029	0.13
lunch	supper	dinner	0.019	0.269
master	owner	servant	0.01	0.108
nation	state	country	0.042	0.345
nature	trail	tree	0.023	0.292
novel	story	book	0.034	0.608
office	doctor	desk	0.014	0.174
opera	tenor	sing	0.021	0.257
pants	clothes	shirt	0.033	0.185
people	world	crowd	0.014	0.155
range	rifle	stove	0.015	0.149
sheet	cover	paper	0.021	0.428
smile	teeth	happy	0.061	0.333

station	radio	gas	0.067	0.241
store	general	buy	0.016	0.142
sweat	run	hot	0.021	0.108
tail	rat	cat	0.021	0.11
tea	cup	bag	0.054	0.087
train	plane	track	0.051	0.327
video	film	camera	0.013	0.126
yarn	string	knit	0.031	0.386

Supplementary Table 1. ***Word Pairs and Association Strengths.*** *Note.* A word pair is hidden due to unintended racial insensitivity. We deeply apologize for this oversight in our stimuli. Forward strength measures how closely related the cue word is to the target based on norms from Nelson et al., (2004). For each cue, high relatedness targets have higher forward strengths.

A Dual-Memory Account with Positively Correlated Memory Strengths

We simulated the effect of positively correlated test and study memory strengths in the dual-memory model using a standardized bivariate normal memory strength distribution (i.e., with $\mu = 0$ and $\sigma = 1$ for both study and test memory strength), and a correlation parameter, ρ. The model does not depend on that choice of distribution. Rather, that distribution was selected for simplicity in the simulation. For each participant, study and test memory strengths for each test item were simulated using random variates from the bivariate distribution, and using the participant's observed PC_R value to set the memory strength threshold for correct retrieval. For example, if a given participant had an observed $PC_R = .3$, then the distribution strength threshold value for correct retrieval from both study or test memory was set to a value (in this example, $z = .524$) such that 30% of the item strength distribution was above the threshold (see supplementary Figure 1).

For each participant, 10,000 test items (10,000 random deviates) were simulated to generate a precise estimate for $PC_{T\text{-predicted}}$. In accordance with the model, if either the study

memory or test memory strength (or both) for a simulated item exceeded the correct response threshold, a correct response was recorded (taking a value of 1). Otherwise, the simulated item yielded an incorrect response (taking a value of zero). The mean of those values over all 10,000 simulated items constituted that participant's $PC_{T\text{-predicted}}$.

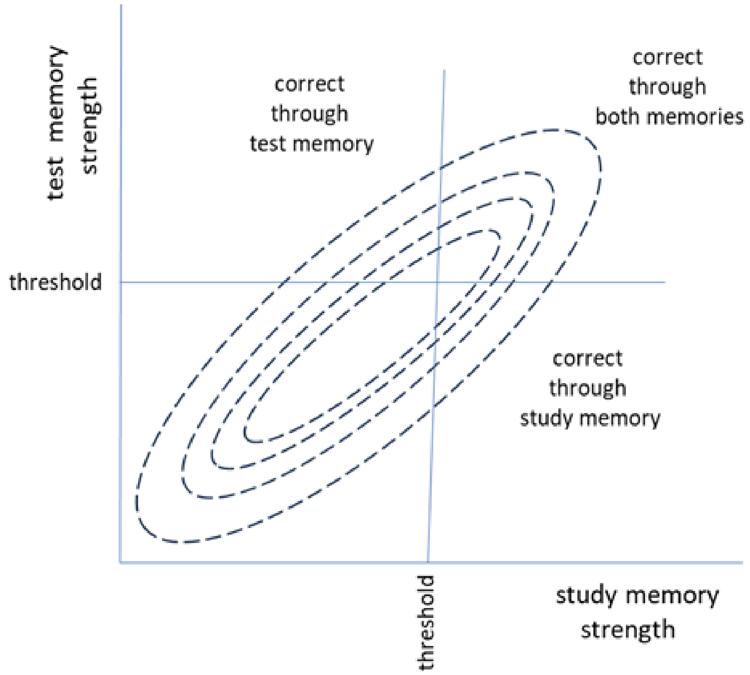

Supplementary Figure 1. **Bivariate Normal Representation of Correlated Study and Test Memory Strengths.** An approximate representation of a positively correlated, standardized bivariate normal distribution for tested items, with random variables study memory strength and test memory strength. The concentric ovals are analogous to contour lines on a topographic map, but in this case represent increasing probability density from edge to center. The identical strength threshold values on the study and test memory strength axes separate items with memory strengths that are below or above that needed for correct responding.

For simplicity, the free parameter ρ was assumed to have the same value for each participant. The value of ρ yielding the smallest absolute deviation between the mean predicted PC_T and mean observed PC_T values was found by iteration through values from zero to one in increments of .001. This *correlated strengths* version of the model can fit the mean PC_T with

arbitrarily high accuracy, and so cannot be tested on that basis. It is not predetermined to fit well to the cumulative distribution of the PC_T, however.

When $\rho = 0$, the simulated prediction for PC_T is identical to that of the independent strengths analytical model (Equation 1), and the ovals in Figure B1 become circles. At the other extreme, where $\rho = 1$, the joint distribution as represented in Figure B1 reduces to a straight line, and there are no longer regions in which either study memory or test memory, but not both, yield the correct response. Rather, for each item, both study and test memory are either above threshold or below threshold, so there is no performance advantage to having both study and test memory retrieval routes for tested items. Hence, the predicted TE is zero. As ρ goes from zero to one, the probabilities corresponding to the regions of the distribution in which correct retrieval occurs through only one of the two retrieval routes becomes progressively smaller relative to the probability that retrieval occurs through both routes, and hence the predicted TE becomes progressively smaller. The value of ρ yielding the best fit to the mean observed PC_T was .334.

To further illustrate the general characteristics of the correlated strengths model, we plotted the cumulative distribution predictions, relative those of the independent strengths model, for various values of ρ ranging from zero to one (Supplementary Figure 2). When the study and test memories are perfectly correlated ($\rho = 1$), the predicted TE magnitude is zero across the distribution. As the correlation decreases the predicted TE magnitude increases, reaching the exact level predicted by the independent strengths model when ρ equals zero. For correlations of about .1 or smaller, systematic deviations between the correlated and independent strengths versions of the model are small and may be difficult to detect experimentally.

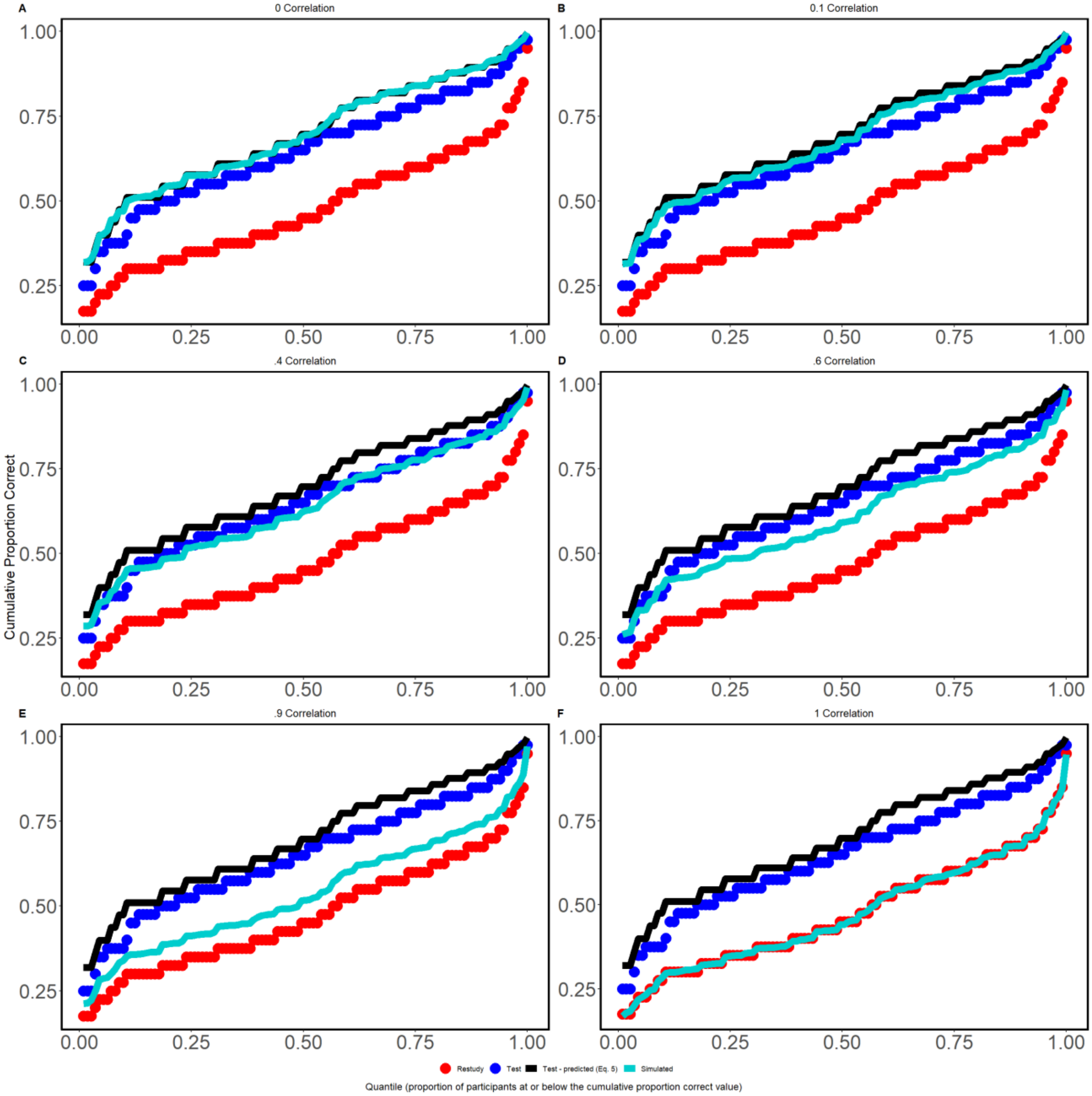

Supplementary Figure 2. **Correlated Strengths Model Fits Across Ranges of ρ.** Cumulative distribution plots of real and simulated data. Visually, there is an obvious "dose-response" relationship: as the study and test memory become more correlated, the reduction in the TE is progressively reduced. The largest reductions appear from .6 to .9 and from .9 to 1.
